# Supplementary material for: Safety of hyperbaric oxygen therapy in patients with heart failure: A retrospective cohort study
Source: PLoS One. 2024 Feb 8;19(2):e0293484. doi: 10.1371/journal.pone.0293484 (PMC10852233; doi:10.1371/journal.pone.0293484)
Supplement: S1 Table — (DOCX) [file pone.0293484.s001.docx]

**S1 Table. Health Canada Approved Indications for Hyperbaric Oxygen Therapy**

| Air or Gas Embolism |
| --- |
| Carbon Monoxide Poisoning |
| Gas Gangrene |
| Compartment Syndrome, Crush Injury, or other Traumatic Ischemias |
| Decompression Sickness |
| Non-Healing Wounds |
| Severe Anemia |
| Intracranial Abscess |
| Necrotizing Soft Tissue Infection |
| Osteomyelitis |
| Delayed Radiation Injury |
| Compromised Skin Grafts and Flaps |
| Thermal Burn Injury |
| Idiopathic Sudden Sensorineural Hearing Loss |
