# Supplementary figures and images for: Safety of hyperbaric oxygen therapy in patients with heart failure: A retrospective cohort study
Source: PLoS One. 2024 Feb 8;19(2):e0293484. doi: 10.1371/journal.pone.0293484 (PMC10852233; doi:10.1371/journal.pone.0293484)

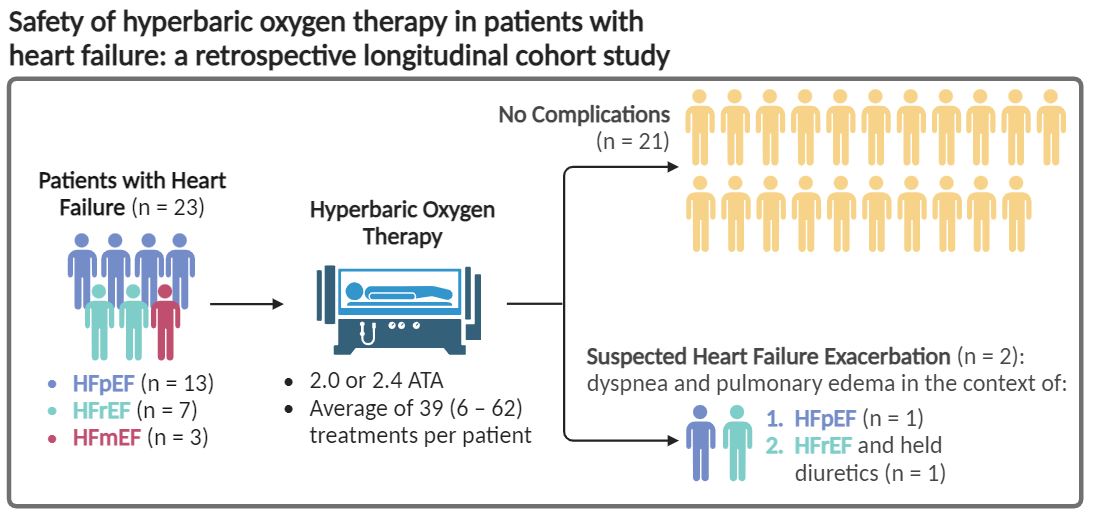

Supplement: S1 Graphical abstract — (JPG) [file pone.0293484.s002.JPG]
