# Supplementary material for: Safety of hyperbaric oxygen therapy in patients with heart failure: A retrospective cohort study
Source: PLoS One. 2024 Feb 8;19(2):e0293484. doi: 10.1371/journal.pone.0293484 (PMC10852233; doi:10.1371/journal.pone.0293484)
Supplement: Attachment — Submitted filename: Revision academic editor2_HBO in HF 2023.docx [file pone.0293484.s003.docx]

ARTICLE SUBMISSION – Revision, academic editor

*PLOS ONE* Editorial Office

**Academic Editor**: Yashendra Sethi

November 9^th^, 2023

Re: Safety of hyperbaric oxygen therapy in patients with heart failure: a retrospective longitudinal cohort study.

Dear Academic Editor,

Thank you for your comments and suggestions.

Please find below the answer to your comments:

When submitting your revision, we need you to address these additional requirements.

1. Please ensure that your manuscript meets PLOS ONE's style requirements, including those for file naming. The PLOS ONE style templates can be found at

**Answer:** This has been checked

2. In your Data Availability statement, you have not specified where the minimal data set underlying the results described in your manuscript can be found. PLOS defines a study's minimal data set as the underlying data used to reach the conclusions drawn in the manuscript and any additional data required to replicate the reported study findings in their entirety. All PLOS journals require that the minimal data set be made fully available. For more information about our data policy, please see [http://journals.plos.org/plosone/s/data-availability](https://urldefense.com/v3/__http:/journals.plos.org/plosone/s/data-availability__;!!CjcC7IQ!LsNo2HqbBfEB5H_GABds-tbeOqzHDqUaSrZJS_JaFPayM5IWVtuPWKzOo0Xj_4T1_hWaYwcPxP72kq-WpXg$). ""Upon re-submitting your revised manuscript, please upload your study’s minimal underlying data set as either Supporting Information files or to a stable, public repository and include the relevant URLs, DOIs, or accession numbers within your revised cover letter. For a list of acceptable repositories, please see [http://journals.plos.org/plosone/s/data-availability#loc-recommended-repositories](https://urldefense.com/v3/__http:/journals.plos.org/plosone/s/data-availability*loc-recommended-repositories__;Iw!!CjcC7IQ!LsNo2HqbBfEB5H_GABds-tbeOqzHDqUaSrZJS_JaFPayM5IWVtuPWKzOo0Xj_4T1_hWaYwcPxP72OZSc8B4$). Any potentially identifying patient information must be fully anonymized. Important: If there are ethical or legal restrictions to sharing your data publicly, please explain these restrictions in detail. Please see our guidelines for more information on what we consider unacceptable restrictions to publicly sharing data: [http://journals.plos.org/plosone/s/data-availability#loc-unacceptable-data-access-restrictions](https://urldefense.com/v3/__http:/journals.plos.org/plosone/s/data-availability*loc-unacceptable-data-access-restrictions__;Iw!!CjcC7IQ!LsNo2HqbBfEB5H_GABds-tbeOqzHDqUaSrZJS_JaFPayM5IWVtuPWKzOo0Xj_4T1_hWaYwcPxP72Hs-LDNc$). Note that it is not acceptable for the authors to be the sole named individuals responsible for ensuring data access. We will update your Data Availability statement to reflect the information you provide in your cover letter.

**Answer:** The source data from our study cannot be shared publicly, in full, because of an ethical restriction levied by our institutional research ethics board. This is because the data contains sensitive information from patient’s medical charts (e.g., birth dates and personal health information). Taken together, this information may allow for the identification of individual study participants. We offer that an anonymized minimal data set can be prepared in aggregate and made available upon reasonable request via email to the study’s first author (simone.schiavo@uhn.ca) or the Hyperbaric Medicine Unit, Toronto General Hospital, Toronto, Ontario, Canada ([hyperbaricmedicineunit@uhn.ca](mailto:hyperbaricmedicineunit@uhn.ca)).

3. Please include your full ethics statement in the ‘Methods’ section of your manuscript file. In your statement, please include the full name of the IRB or ethics committee who approved or waived your study, as well as whether or not you obtained informed written or verbal consent. If consent was waived for your study, please include this information in your statement as well.

**Answer**: The following full Ethic statement is included in the Methods section, and it is inclusive of the required comments: *“This is a retrospective longitudinal cohort study of patients with HF who underwent elective HBOT between June 2018 and December 2020 in two Hyperbaric Medicine Centers in Ontario, Canada (Toronto General Hospital, Toronto; Rouge Valley Hyperbaric Medical Centre, Scarborough). Institutional Research Ethics Board approvals (CAPCR ID: 19-5081.1; IRB ID:2023-3194-14092-4) were obtained for study team members to collect data from medical records (last access to data on March 31, 2023; all authors but one (SS) did not have access to information that could identify individual participants during or after data collection).”*

4. Please review your reference list to ensure that it is complete and correct. If you have cited papers that have been retracted, please include the rationale for doing so in the manuscript text, or remove these references and replace them with relevant current references. Any changes to the reference list should be mentioned in the rebuttal letter that accompanies your revised manuscript. If you need to cite a retracted article, indicate the article’s retracted status in the References list and also include a citation and full reference for the retraction notice.

**Answer:** Thank you for the comment; the reference list has been updated based on the journal style file “PLoS” recommended by, and available at, your journal website. No changes to the references other than reference number 1 that has been updated with the most recent one (book reference; newer edition of the book (15^th^ 2023) rather than the previous (14^th^ edition 2019)). This change, due to technical issues with the Reference Software cannot be flagged with “track changes”, however we highlighted it I yellow to flag the change compared to the previous version of the manuscript.

Additional Editor Comments

5. In your title, justify the use of retrospective review or kindly replace with retrospective longitudinal cohort study.

**Answer:** Thanks for the comment; we have reviewed our title as suggested and changed it accordingly.

6. Please support your study with some digrammatic representation or summary as you may deem fit, it will allow readers a better understanding and attaract more viewership.

**Answer:** This is a valuable suggestion, and we have created and added a diagrammatic representation of the study; this has been added as Supplementary Material “S2 Graphical Abstract”.

7. Revise methods and conclusions section of your abstract to be more clear.

**Answer:** This has been checked and we feel the division in sub-categories in Methods adds clarity to the section. We all revised the conclusions and agreed they represent the authors’ conclusions.

8. If possible, please upload a file showing your changes either highlighted or using track changes.  This should be uploaded as a Revised Manuscript w/tracked changes, file type.

**Answer:** This has been checked and corrected.

9. Please amend the title either on the online submission form or in your manuscript so that they are identical.

**Answer:** This has been checked and corrected.

We thank you for your time and consideration, and remain available for any further clarification or detail if needed.

Sincerely,

Simone Schiavo, MD, MSc

Assistant Professor of Anesthesia, University of Toronto.

Staff Anesthesiologist, Department of Anesthesia, Toronto Western/Toronto General Hospital, UHN.

200 Elizabeth Street, G PMB 106E, Toronto, ON, M5G2C4, Canada

Email: simone.schiavo@uhn.ca

Tel TGH: 416-340-4800 ext. 3598 - Tel TWH: 416-6035800 ext. 5118 - Fax 416-340-4481

Cell: 647-687-8222
