# Supplementary material for: Safety of hyperbaric oxygen therapy in patients with heart failure: A retrospective cohort study
Source: PLoS One. 2024 Feb 8;19(2):e0293484. doi: 10.1371/journal.pone.0293484 (PMC10852233; doi:10.1371/journal.pone.0293484)
Supplement: Attachment — Submitted filename: Revision reviewers_HBO in HF 2023 - final.docx [file pone.0293484.s004.docx]

ARTICLE SUBMISSION – Revision, reviewers

*PLOS ONE* Editorial Office

**Reviewers #1 and #2**

**Academic Editor**: Yashendra Sethi

December 16^th^, 2023

Re: Safety of hyperbaric oxygen therapy in patients with heart failure: a retrospective cohort study.

Dear Reviewers,

Thank you for your comments and suggestions. We have addressed these in our revised manuscript, as outlined point-by-point below:

Reviewer #1

The study delves into a crucial issue that could significantly impact our capacity to administer HBOT to deserving patients who might otherwise be excluded. While it is well designed and well written, there are a few issues that should be addressed before its publication:

**Response:** Thank you for the positive feedback and for all the following valuable comments.

1. Line 64: “HBOT increases cardiovascular afterload, with associated increases in systolic and mean arterial blood pressure (BP), while cardiac output (CO) decreases due primarily to a decrease in heart rate (HR).” And line 73: “Numerous mechanisms for the effect of HBOT on CO have been suggested although this effect predominately results from HBOT-induced vasoconstriction, the physiological protective response to extremely high arterial partial pressures of oxygen”. Please consider merging these sentences for more coherent explanation regarding HBOT’s effect on CO:high oxygen level (pressure?) vasoconstriction increases cardiovascular afterload…

**Response:** Thank you for the suggestion; the sentences have now been merged for added clarity (line 64-68).

2. Line 85: Please consider omitting “longitudinal”. Retrospective cohort studies are always 'longitudinal,' because they examine health outcomes over a span of time.

**Response:** Indeed, this is an excellent point. Both the title and line 85 (now line 89) have been changed accordingly. Of note, the original submission did not include the term “longitudinal”, that was added as per the academic editor’s request in order to proceed to the reviewers’ phase.

3. Line 109: “*We included all patients 18 years of age or older with a history of HF”.* As listed in their medical charts? based on baseline echocardiography? Based on their symptoms history? Please be more specific.

**Response**: The definition of history of HF has been clarified in the revised text, per this suggestion (line 115).

4. Line 125: How did you provide air breaks in a mono-place chamber? Please describe the technique. In a multi-place chamber, air breaks are accomplished simply by removing the mask, but in a mono-place chamber, a complete exchange of the chamber’s gas may be required.

**Response:** Thank you for the comment. The performance of an air break inside a mono-place chamber has been the subject of considerable discussion. In short, while a complete exchange of the chamber gas is technically feasible (e.g., in the event of emergency CNS toxicity), it is not doable nor practical for every elective patient. Instead, it is an industry standard to use air break assemblies that are installed on the monoplace chamber itself. These assemblies provide air (FiO2 21%) delivered directly to the patient within the chamber—in our institution, via a non-rebreather face mask with a high flow (60 l/min). While this does not guarantee a strict 21% FiO2, it is a close surrogate and currently accepted as the most feasible and effective method to deliver an air break inside a monoplace chamber. See also: https://learn.hyperbaricmedicine.com/wp-content/uploads/2016/10/Sheffield-R-Air-break-assemblies-_101016.pdf <https://learn.hyperbaricmedicine.com/wp-content/uploads/2016/10/Sheffield-R-Air-break-assemblies-_101016.pdf> , and [Weaver LK. Operational use and patient care in the monoplace hyperbaric chamber. Respir Care Clin N Am. 1999 Mar;5(1):51-92. PMID: 10205813.] We added this information in the lines 130-133.

5. Line 129: *Standard monitoring included measurements of systolic (SAP), diastolic (DAP), and mean (MAP) blood pressure (BP), heart rate (HR), and peripheral oxygen saturation (SpO2) assessed during a five-minute period preceding and following each HBOT session*. Consider providing data regarding the blood pressure of the study cohort, including baseline values and the mean change from baseline following HBOT. Were there any changes in BP during the index HBOT session for subjects who developed HF exacerbation?

**Response:** Thank you for the comment. The BP changes for the second patient who developed HF symptoms were reported (line 217, was line 205), and we added the BP changes for the first case (line 201). An in-depth analysis of BP changes before and after HBO in a cohort of elective patients undergoing HBOT has been previously published by our group *[Schiavo S, Djaiani C, DeBacker J, Albertini L, Santa Mina D, Buryk-Iggers S, De Moraes MV, Kanj M, Katznelson R. Magnitude and Clinical Predictors of Blood Pressure Changes in Patients Undergoing Hyperbaric Oxygen Therapy: A Retrospective Study. Int J Environ Res Public Health. 2020 Oct 19;17(20):7586. Doi: 10.3390/ijerph17207586. PMID: 33086495; PMCID: PMC7589597*]. The data of the current HF cohort do not differ significantly, and so thoroughly detailing blood pressure in the present cohort would not contribute further novelty. However, given the excellent consideration the reviewer raised in this comment on the clinical implications (see below), we have also added a comment about BP changes in Discussion – Clinical Implications (line 287).

6. Clinical implications: Describe your practice following BP evaluation before treatment. Do you exclude patients with uncontrolled hypertension from entering the session? Such a practice may impact CHF exacerbation rates and should be recommended if applicable.

**Response:** This is a very valuable suggestion, and we have added a description of standard practice at our center (line 285).

7. Please add title to the table. Are the mentioned indications approved by Health Canada? By FDA?

**Response:** The reviewer raises a good point; the Table Title and the required information were described in the Manuscript in the section Supporting information, after the References. Due to the journal uploading website, these were lost in the actual Table image. We are grateful for their attention to this detail, and we will double check with the Editor that these will be included in the final version, if accepted.

Reviewer #2

1. Article is an excellent article. worth publishing. Any role of prophylactic diuretics and NT PRO BNP measurements before starting the HBOT?? Superb article. Congratulations..

**Response:** We thank the reviewer for the very positive comments. Both points are indeed matters of ongoing research and significant interest at our center. Thank you again.

We thank you for your time and consideration, and remain available for any further clarification or detail if needed.

Sincerely,

Simone Schiavo, MD, MSc

Assistant Professor of Anesthesia, University of Toronto.

Staff Anesthesiologist, Department of Anesthesia, Toronto Western/Toronto General Hospital, UHN.

200 Elizabeth Street, G PMB 106E, Toronto, ON, M5G2C4, Canada

Email: simone.schiavo@uhn.ca

Tel TGH: 416-340-4800 ext. 3598 - Tel TWH: 416-6035800 ext. 5118 - Fax 416-340-4481

Cell: 647-687-8222
